# Convergence of sensory and limbic noxious input into the anterior insula and the emergence of pain from nociception

**DOI:** 10.1038/s41598-018-31781-z

**Published:** 2018-09-06

**Authors:** Hélène Bastuji, Maud Frot, Caroline Perchet, Koichi Hagiwara, Luis Garcia-Larrea

**Affiliations:** 1 Central Integration of Pain (NeuroPain) Lab - Lyon Neuroscience Research Center, INSERM U1028; CNRS. UMR5292, Université Claude Bernard, Bron, F-69677 France; 2 Unité d’Hypnologie, Service de Neurologie Fonctionnelle et d’Épileptologie, Hôpital Neurologique, Hospices Civils de Lyon, Bron, F-69677 France; 3 Centre d’évaluation et de traitement de la douleur, Hôpital Neurologique, Lyon, France

## Abstract

Two parallel di-synaptic routes convey nociceptive input to the telencephalon: the spino-thalamic system projecting principally to the posterior insula, and the spino-parabrachial pathway reaching the amygdalar nucleus. Interplay between the two systems underlies the sensory and emotional aspects of pain, and was explored here in humans with simultaneous recordings from the amygdala, posterior and anterior insulae. Onsets of thermo-nociceptive responses were virtually identical in the posterior insula and the amygdalar complex, but no significant functional connectivity was detected between them using coherence analysis. Anterior insular sectors responded with ~30 ms delay relative to both the posterior insula and the amygdala. While intra-insular functional correlation was significant during the whole analysis period, coherence between the anterior insula and the amygdala became significant after 700 ms of processing. Phase lags indicated information transfer initially directed from the amygdalar complex to the insula. Parallel but independent activation of sensory and limbic nociceptive networks appear to converge in the anterior insula in less than one second. While the anterior insula is often considered as providing input into the limbic system, our results underscore its reverse role, i.e., receiving and integrating very rapidly limbic with sensory input, to initiate a perceptual decision on the stimulus ‘painfulness’.

## Introduction

Pain is a complex experience involving sensory, cognitive and motivational components. Such diversity is supported by a variety of ascending systems conveying nociceptive information from the spinal cord to the telencephalon, of which the two main disynaptic routes are the spino-thalamo-cortical (STC) and the spino-parabrachial-amygdalar pathways. After relay in multiple nuclei of the posterior thalamus^1,2^, the STC pathway in primates targets three main cortical regions involved in sensorimotor integration and attentional drive, namely the posterior insula, the medial parietal operculum, and the mid-cingulate cortex, with less extensive projections to primary sensory and motor areas^3,4^. The spino-parabrachial route, on the other hand, reaches the limbic system via the amygdalar complex, especially its central nucleus, and participates to the triggering of autonomic responses and the elaboration of affective components of pain^5–7^ Recent data from intracranial recordings in humans have suggested that the onset latencies of amygdalar responses are virtually identical to those in posterior insular and mid-cingulate cortices^8^, and hence that the sensory, orienting and affective aspects triggered by nociceptive input are processed in parallel rather than sequentially^9^.

The human posterior insula is able to encode sensory aspects of nociceptive input including intensity, somatotopy and sensory sub-modality^10–13^. Intracranial recordings suggest a rapid information flow from posterior to anterior insula^14^ –the latter being an agranular cortex implicated in integrative aspects of pain, including affective and visceral reactions associated to the painful sensation^15,16^. The connectivity patterns of posterior and anterior insulae differ substantially^17,18^. The anterior portions of the insula, together with the temporal pole and lateral orbitofrontal cortex, were termed “paralimbic” because of their extensive reciprocal interconnections with limbic structures in rhesus monkeys^19–21^.

The existence of parallel disynaptic pathways conveying input to sensory and limbic structures suggests that they both may have simultaneous access to noxious information. Such dual source must be incorporated into higher-order networks in order to build the unified subjective perception qualified as “pain”. It has been proposed that convergence of multimodal input to the most anterior portions of the insula contributes to self-recognition, emotional awareness and the building of consciousness^22^; the anterior insula may therefore represent a core system (a ‘hub’) integrating affective and sensory information, and contributing to the building up of a unified but multi-faceted subjective sensation.

Here we tested the above hypothesis by analysing nociceptive-specific evoked potentials and functional connectivity in 10 epileptic patients with electrodes simultaneously implanted in the posterior and anterior insular sectors, as well as in the amygdalar nucleus. Such exceptional access to responses in the three regions allowed analysing both response timing and functional inter-areal relationships via phase-coherence, and generated a comprehensive image of the activities of the three structures. The results suggest that, during the first second that follows a noxious stimulus, an initial parallel and uncorrelated nociceptive processing in sensory and limbic systems is rapidly followed by a functional convergence of both toward the anterior insula.

## Results

### Timing and amplitude of insular and amygdalar nociceptive responses

**Figure 1:**
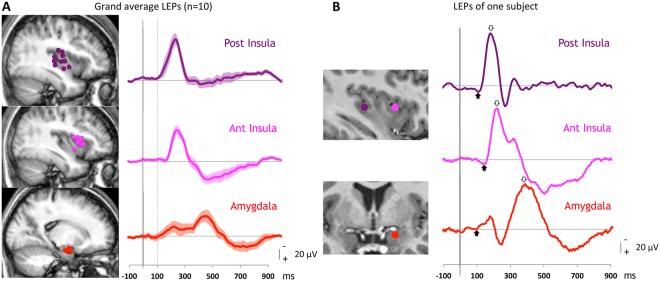
Laser evoked potentials (LEPs) recorded in the posterior and anterior insulae and the amygdalar nucleus. (**A**) All subjects. Left: Recording contact locations in each area plotted on mean sagittal slices obtained from all the patients MRI; top: posterior insula (magenta dots); middle: anterior insula (pink dots); bottom: amygdala (red dots). Right: Grand averages (+/− SEM) of responses obtained in each area (in referential mode). The dotted vertical line represents the onset of the posterior insular LEPs. (**B**) One representative subject. Left: Recording contact locations represented on the patient’s MRI with colour circles in the posterior and anterior insulae (top, sagittal view)) and amygdala (bottom, coronal view). Right: LEPs obtained in each of the three areas (in referential mode). The arrows indicate the onset and peaks of the responses. Note that the onsets of the responses occur simultaneously in the posterior insula and amygdala and later in anterior insula, while the highest peak of the amygdalar response occurs later than the two others.

Grand averaged responses from the 3 areas together with responses of a representative patient are illustrated in Fig. 1A and B. While the posterior and anterior insular potentials were similar in shape, the morphology of the amygdalar response was different from those in the insulae, lasting much longer and often showing two consecutive peaks of which the second one was always predominant.

**Figure 2:**
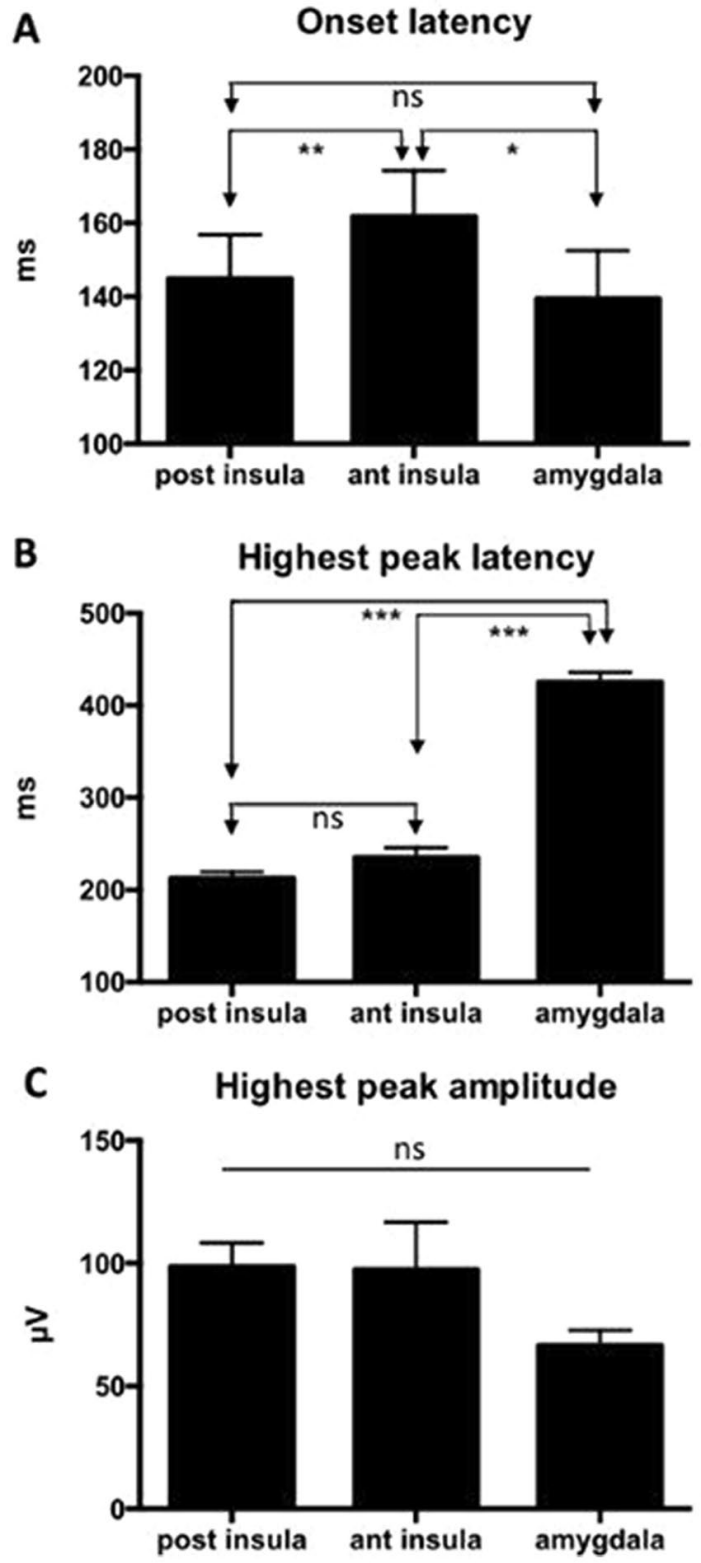
Latency and amplitude histograms of LEPs recorded within the posterior insula, anterior insula and amygdala. Error bars: ±SEM. (**A**) The onset latencies in posterior insula and amygdala were not significantly different, while both responded with significantly shorter onset than the anterior insula. (**B**) The main peak latency was delayed in the amygdala as compared to both posterior and anterior insulae. The anterior insular response peaked slightly later than its posterior counterpart but the difference was not significant. (**C**) There was no significant difference of the amplitude of the response between the 3 areas.

One-way ANOVA demonstrated a significant onset latency difference among regions [F^2,9^ = 9.96; P = 0.0045]. While onset latencies between posterior insula and amygdala did not differ, they were both significantly shorter than latencies in the anterior insula (Holm-Sidak, p = 0.0025 and p = 0.04, corrected) (Fig. 2A). The main peak latencies also differed significantly among the three cortical areas [F^2,9^ = 120.2; P < 0.0001], the latencies of culmination being delayed in the amygdala as compared to both posterior and anterior insulae (both: p < 0.0001). The anterior insular response peaked later than its posterior counterpart, but the difference only showed a trend toward significance (corrected p = 0.088) (Fig. 2B). The amplitude of the response at its highest peak did not differ among the three areas [F^2,9^ = 2.24; P = 0.157] (Fig. 2C).

### Functional connectivity analyses

**Figure 3:**
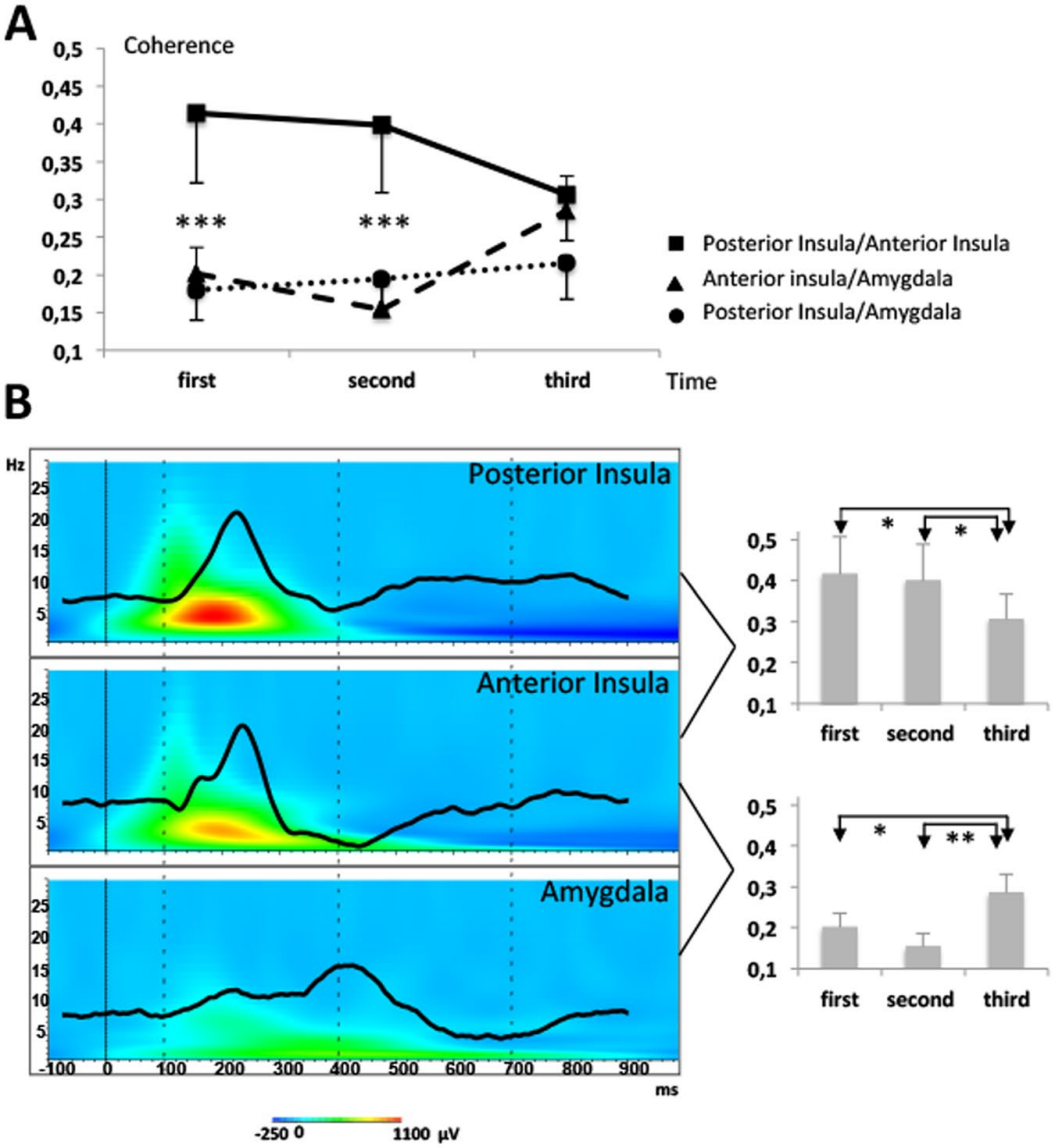
(**A**) Interaction between cortical areas and time, on spectral coherence values. Mean phase spectral coherence values of EEG signal between each combination of areas and three time windows (100–400 ms; 400–700 ms; 700–1000 ms). The significant differences between both insulae and the two other pairs were p < 0.0001 (***). (**B**) (Left panel). Grand averages of LEPs in posterior insula (top), anterior insula (middle) and amygdala (bottom) superimposed on their representation on a time-frequency axis. Dotted lines indicate the 3 time windows chosen for coherence analysis. (**B**) (Right panel). Mean phase spectral coherence values between both insulae (top) and between anterior insula and amygdala (bottom) during the 3 time windows. Significant differences on post hoc analyses are indicated: *p < 0.05 and **p < 0.01.

Three-way ANOVA on spectral coherence values showed no significant main effect of cortical area [F^2,9^ = 2.12; *p* = 0.14], time window [F^2,9^ = 0.26; *p* = 0.77] or frequency band [F^1,9^ = 0.74; *p* = 0.40]. There was, however, a significant interaction between cortical area and time-window [F^4,18^ = 3.03; *p* = 0.025], due to a significant decline of intra-insular coherence values in the last analysis period (700–1000 ms), in coincidence with enhanced coherence between the amygdala and the *anterior* insula during the same period (Fig. 3A).

**Figure 4:**
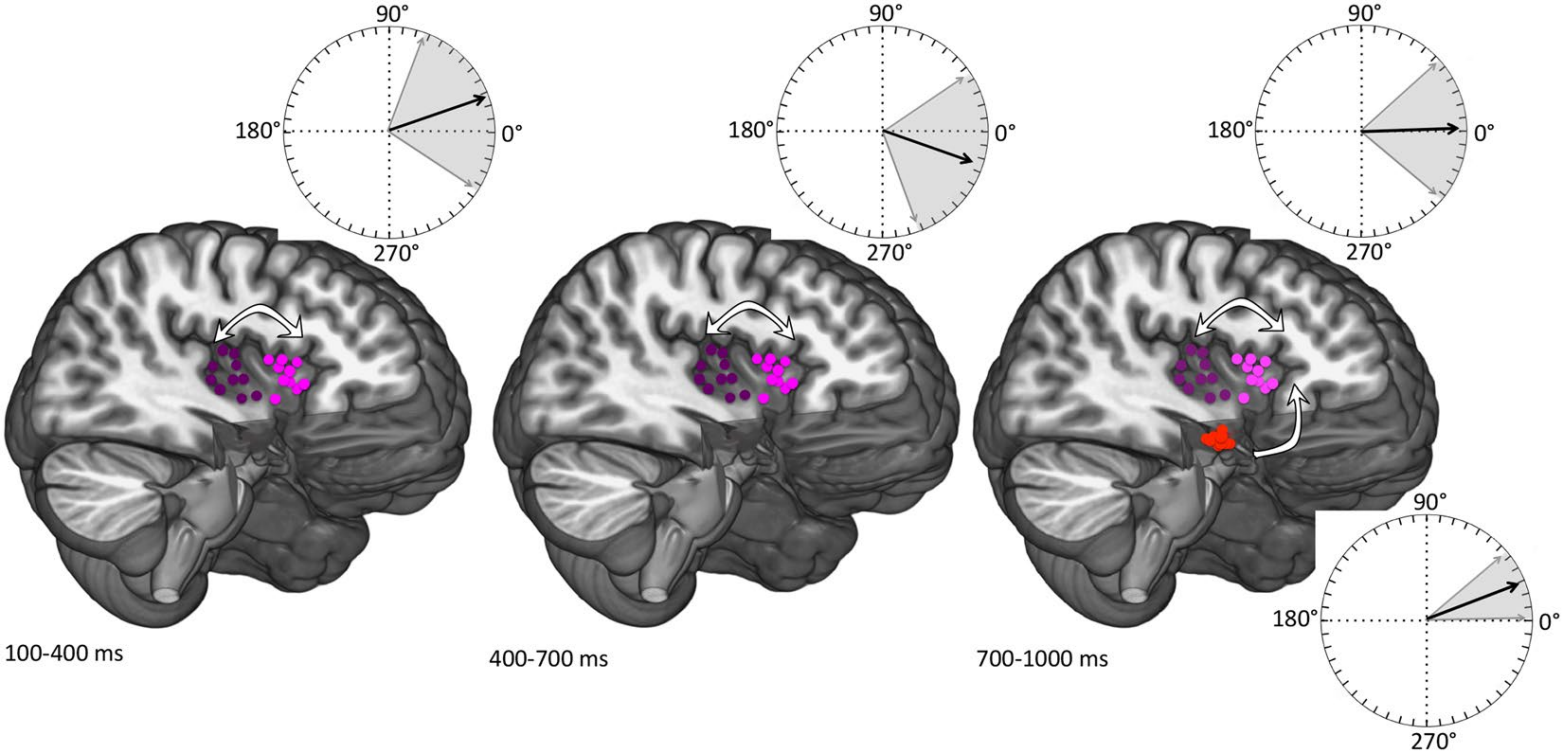
Mean phase delays with 95% confidence intervals), represented on trigonometric circles, between the pairs of structures where mean coherence values were significant (>0.2) (posterior/anterior insulae in the 3 time windows and anterior insula/amygdala in the last time window). Contact locations in each brain area are projected on the averaged MRI of all patients. Posterior insula (magenta dots); anterior insula (pink dots); amygdala (red dots). Arrows indicate the estimated functional directionality between structures at each period of time.

Figure 4 illustrates the mean phase delays and confidence intervals between the pairs of structures where coherence values were significant (>0.2). For each patient, phase shifts were computed at the frequency exhibiting the highest coherence, which was always between 5 and 10 Hz (mean 7.9 ± 1.7 Hz). As shown in Fig. 4 (top diagrams), the intra-insular average phase lags (±SEM) were positive during the initial time window (19.2 ± 26.7) and negative during the second (−17.8 ± 21.4), in favour of a change of directionality of information transfer; however, inter-individual differences were very important and confidence intervals always crossed the zero line (Fig. 4, upper part).

In contrast with this, phase lags between the amygdala and the anterior insula in the 700–1000 time window were concentrated on the upper right quadrant of the phase circle (Fig. 4, lower right). The average phase lag (±SEM) was 21 ± 10° in the ‘amygdala toward insula’ direction, and 339 ± 10° in the insula-to-amygdala opposite sense. Once transformed into milliseconds, this yielded a mean time lag of 8.1 ± 3 ms between amygdalar and anterior insular oscillations if the amygdala was considered to lead, and of 118 ± 3 ms if the insula was leading.

## Discussion

### Parallel but independent activation of sensory and limbic networks

Field responses to nociceptive input confirmed virtually identical onset latencies in the posterior sensory insula and the amygdalar nucleus^8^. Comparable onset times of activation in these two regions suggest that one was not triggered by the other, but rather that nociceptive input reached simultaneously the posterior insula and the amygdala, respectively via the spino-thalamo-cortical and the spino-parabrachial-amygdalar pathways^5,23^. Functional independence of nociceptive processing in the amygdala and posterior insula was also supported by the lack of significant spectral coherence between the two regions during the whole analysis window (Fig. 3). This contrasted with the significant coherence levels rapidly observed between the posterior and anterior insular sub-regions, and suggests that, at least during the initial stages of processing, the handling of nociceptive input in sensory and limbic targets remained independent and uncorrelated. Although early tracing studies reported anatomical projections between these two areas in monkeys^20^, the ‘posterior’ insular injections in these studies incorporated dysgranular sections anterior and ventral to the posterior insula, thus greatly limiting the injection selectivity (see Figs 3 and 6 in^20^). More recent studies in monkeys^24^ and humans^25^ have suggested that activity in the posterior insula, while heavily coupled with that of somato-motor areas, is uncorrelated with that of limbic regions, and in particular the amygdala. Also, a recent analysis of structural connectivity of insular subdivisions in humans failed to disclose anatomical connections between the posterior insula and the amygdala^26^.

### Spectral coherence reflects rapid bi-directional intra-insular communication

The posterior and anterior sections of the insula showed the highest levels of functional relationship from the very beginning of the response, declining only during the last period of the analysis window. Anatomical and functional interconnections between the posterior and anterior insula have been consistently reported in both monkeys and humans^17,21,26–28^. Although intra-insular communication is bi-directional, initial information transfer after a somatic stimulus is likely to occur from the posterior sensory sectors toward the more anterior and integrative insular divisions, and indeed in our patients the response in the posterior insula started earlier than in its anterior counterpart (Fig. 2). The study of phase relationships did not allow, however, establishing a clear pattern of directionality, since phase angles between signals were highly dispersed within the trigonometric circle (Fig. 4). Indeed, although the average phase lags were consistent with an initial postero-anterior flux (+19.2°) with subsequent reversal (−17.8°), dispersion of individual values yielded confidence intervals too large to allow reliable interpretation (Fig. 4, upper part). While the initial intra-insular coherence is likely to depend on a rapid flux of information from posterior to anterior sectors, most intra-insular connections in humans were found to be reciprocal, and a previous report did not find a clear directionality^28^. The initial postero-anterior flux may have been concealed by a rapid flow back, thereby blurring any consistent directionality.

### Does sensory and limbic information converge in the anterior insula?

Phase relationships between anterior insula and amygdala showed a much more consistent pattern than the one observed for intra-insular relations. Phase angles were mostly concentrated within the upper right quadrant of the trigonometric circle, with a mean phase difference of 21° (~8 ms) between amygdalar and anterior insular activities (Fig. 4). Calculating time shifts from phase angles entails an inherent ambiguity, since 360° can always be added to or subtracted from a phase value^29^. Thus, a lead of 8 ms if the amygdala leads the insula can also correspond to a lag of 118 ms if we consider that the insula is leading. In mammals, latency delays between contiguous cortical regions or homologous areas did not exceed 20 ms in most reported studies. Average delay between S1 and S2 in cats was 13 ms^30^, and mean latencies to activate area 17 from areas 18 and 19 were 6.1 and 10.4 ms respectively^31^. In humans, inter-hemispheric transmission between homologous S2 areas was reported to be of ~15 ms^32,33^, and direct electrical stimulation in epileptic patients estimated conduction times between insula and amygdala at 28 ± 3 ms^34^. Despite substantial variation of individual results around these averages^30,35^, published latency histograms show that 40–60% of cortico-cortical connections have latencies less than 10 ms^30,36,37^. In the present case the mean time lag around 8 ms if the amygdala is leading the insula appears physiologically compatible with published intra-cortical delays, whereas the 118 ms lag we would obtain if the insula was leading the amygdala is not. This supports the view that the enhanced coherence between the two structures reflects, at least initially, information transfer from the amygdalar nucleus to the anterior insula.

Insular–amygdalar relationships are reciprocal, their overall functional connectivity is certainly bidirectional^20,34^ and the above results should not be taken as reflecting causality. The anterior insular cortex provides extensive input into the amygdaloid nucleus, while equally extensive amygdalar connexions toward the anterior insula involve the medial, cortical, basal and lateral nuclei^19,20^. The finding that the initial relationship between the two regions may convey information from the amygadala to the insula, and not the opposite, is consistent with the fact that the onset response times of the amygdalar complex preceded those of the anterior insula (Fig. 1). This suggests that the anterior insula represents an area of sensory-limbic convergence, capable of receiving and integrating very rapidly sensory information from its posterior sector with affective input from the amygdala, and hence participates to the initial transformation of cortical nociception into the experience of pain.

Mesulam and Mufson^17^ considered the anterior insula as part of a paralimbic cortex interfacing extrapersonal stimuli and the internal milieu. Detecting stimulus relevance and engaging executive regions for appropriate responses via its extensive output to fronto-cingulate networks is indeed considered as one of its core functions^38,39^. Functional imaging has contributed to establish the anterior insula as a pivotal area integrating systems involved in affect, sensory processing, and general cognition^39–42^. In a comprehensive study of networks coactivated with insular regions, Chang *et al*.^41^ described clear differences between the posterior granular insula (a multimodal zone for sensory input) and the anterior dys- and agranular insula mainly associated with networks involved in emotion, autonomic function, higher cognitive tasks and executive control. Most reports, however, tend to disregard the importance that *afferent* connections may have to determine the ‘functions’ ascribed to this region. Clinical reports have suggested, however, that destruction of connections to the anterior insula, including from medial temporo-limbic cortex, may impair the recognition of emotions to a greater extent than lesions of the anterior insula itself^43^.

### Limitations of the study

We analysed cortical responses to nociceptive stimuli exclusively, and therefore the results cannot be generalized to input from other somatosensory subsystems. Also, although the time lags obtained here suggest direct cortico-cortical connections, functional links through phase-coherence analysis do not prove *causal* influences from one structure over another^44^. The amplitude gradient between the critical recording contact and its neighbours indicates that the activities analysed here were very close to the actual sources, in the latero-medial axis (see Fig. S1). However, we cannot exclude some volume-conducted activity in the dorsal-ventral axis, especially within the anterior insula where this axis is maximal. Thus, whether our recordings mainly reflected activity from antero-dorsal or antero-ventral subsections, mostly connected with the amygdala^26^, goes beyond the localization capacities of this study. Similarly, the spatial resolution of intracranial macroelectrodes used for human recordings does not allow assessing specific subnuclei responsible for amygdalar responses. This might become possible in the future with the development of systems allowing simultaneous recording of local field potentials and single- or multi-unit responses^45^. Finally, confining our analysis to the insular–amygdalar relations is necessarily a simplification, and this work should be progressively expanded to incorporate other structures active during the same and subsequent time spans.

## Conclusion

Evoked field potentials and coherence analyses were consistent with a model of nociceptive integration whereby the spino-thalamic and spino-parabrachial systems reach their respective telencephalic targets in parallel, but with no or very little mutual interaction. In less than one second the pattern of inter-areal coherence changes from an initial predominance of posterior-anterior intra-insular links, to a configuration where the anterior insula becomes similarly interconnected with its posterior sensory sector and with the amygdala. The results point to the anterior insula as an area of sensory-limbic convergence, integrating sensory with emotional input, and hence participating to the transformation of cortical nociception into the experience of pain.

## Methods

### Patients

Ten patients with refractory partial epilepsy were included in the study (7 men, 3 women; mean age 27 years, range 19–51 years). To delineate the extent of the cortical epileptogenic area and to plan a tailored surgical treatment, depth EEG recording electrodes (diameter 0.8 mm; 5–15 recording contacts 2 mm long, inter-contact interval 1.5 mm) were implanted according to the stereotactic technique of Talairach and Bancaud^46^. In agreement with French regulations relative to invasive investigations with direct individual benefit, patients were fully informed about electrode implantation, stereotactic EEG (SEEG), evoked potential recordings, and cortical stimulation procedures to localize the epileptogenic cortical areas and gave their consent. The laser stimulation paradigm was approved by the local and national Ethics Committee (CPP Sud Est IV n° 2006-A00572-49 and Ile de France XI n° 2017-A00464-49). Recordings were conducted after a minimal delay of five days post electrode implantation and none of these patients reported pain symptoms before or after the recording session.

### Anatomical localization of electrode contacts

The localization of the recording contacts was determined using the appropriate slices MR of patient’s brains [MRIcron® software] (See SI). Intracortical electrode contacts were mapped to the standard stereotaxic space (Montreal Neurological Institute, MNI) by processing MRI data with Statistical Parametric Mapping (SPM12). Then, the localization of electrodes was performed directly on the MR of the patients, and using the human atlas of the insula^47^ for the exact localization of the contacts within the insula.

### Nociceptive-specific laser stimulation

Radiant nociceptive heat pulses of 5 ms duration were delivered with a Nd:YAP-laser (Yttrium Aluminium Perovskite; wavelength 1.34 µm; El.En.®, Florence, Italy). Nociceptive thresholds to A-delta stimuli were determined as the minimal laser energy producing a pricking sensation, compared to “pulling a hair” or “receiving a boiling water drop” in at least two out of three stimuli. (see SI).

### Data acquisition and recording procedure

In each patient, two runs of 10–15 stimuli each, at nociceptive threshold, were applied to the skin in the superficial radial nerve territory on the dorsum of the hand contralateral to the side of electrodes implantation. Recordings were performed in common referential mode from 96–128 channels, at a sampling frequency of 256 Hz or 512 Hz, then amplified and band-pass filtered (0.33–128 Hz; −3 dB, 12 dB/octave) (Micromed SAS®, Mâcon, France).

### Electrophysiological data analyses

#### Laser-evoked potentials (LEPs)

The coordinates of the contacts exhibiting the largest responses to laser stimuli are indicated on Table 1, and grand-averaged data showing the amplitude gradient between these and adjacent electrode contacts are illustrated in Figure S1 (supplementary data). Offline analyses, including segmentation of the EEG, selective averaging, time-frequency transforms and spectral coherence analyses were performed using BrainVision® System (Brain Products®, Munich, Germany). Segments presenting contamination by epileptic transient activities or artefacts exceeding 250 µV were rejected from analysis, the rate of rejection being of ~10%. LEP components recorded in the different structures were assessed within a time window of 1 second (100 ms pre- and 900 ms post-stimulus). We measured in each patient (1) the onset and peak latencies of the LEP main component, and (2) its amplitude (from onset to peak). Onsets were defined at the inflection point when amplitude values of the signal differed by two standard deviations from the mean pre-stimulus baseline. Statistical analyses were performed with GraphPad Prism 6 and StatView® softwares. Latencies and amplitudes were submitted to one-way ANOVA with cortical areas as ‘between’ factor, and significance level set at p < 0.05 (Greenhouse-Geisser corrected if needed). Post-hoc tests (Holm-Sidak test corrected for multiple comparisons) were applied in case of significant main effects of ANOVA.

**Table 1:** MNI coordinates (x, y, z) of cortical contacts with the largest LEPs.

Patients	Posterior Insula	Anterior Insula	Amygdala
P1	35, −23, 2	33, 11, 9	16, −3, −22
P2	35, −22, 7	36, 11, 2	25, 2, −22
P3	37, −12, 2	35, 8, 5	24, −2, −21
P4	38, −6, 4	35, 9, 10	15, −2, −19
P5	37, −1, −4	38, 16, 1	15, −6, −20
P6	38, −16, 17	35, 0, 11	18, −3, −20
P7	35, −13, 15	37, 7, 4	18, −9, −21
P8	36, −10, 12	35, 5, 7	26, −7, −21
P9	38, −21, −2	37, 2, −7	19, −4, −24
P10	35, −8, −8	35, 5, 11	22, 1, −28
Mean	**36**, −**13**, **5**	**36**, **8**, **7**	**20**, −**3**, −**22**
SD	**1**, **7**, **8**	**1**, **7**, **5**	**4**, **3**, **3**

#### Functional connectivity measures

Intra-insular and insula-amygdala functional relationships were assessed using EEG phase-coherence between each pairs of areas (posterior insula-anterior insula, posterior insula-amygdala and anterior insula-amygdala). Phase coherence was computed after Fast Fourier Transform of the signal for each spectral band power (delta: 1–3 Hz, theta: 4–7 Hz, alpha: 8–12 Hz, beta: 13–29 Hz). The analysis was performed within three post-stimulus time windows: 100–400 ms, 400–700 ms and 700–1000 ms^8^. Phase coherence values were computed as the quotient between correlation and autocorrelation for each frequency and each channel pair, and underwent Fisher’s z-transformation before statistical analysis. Phase values were unwrapped (phase as a continuous signal across frequency bins), then phase angles were transformed into milliseconds. Coherence values were grouped as ‘low frequencies’ (delta + theta bands, 1–7 Hz) and ‘high-frequencies’ (alpha + beta bands, 8–29 Hz). To determine whether a given level of coherence between two regions was above noise, its statistical significance was estimated by contrasting with random levels of coherence, obtained from recordings where the amplitude levels of one of the time series was randomly reordered^48^. Therefore, coherence levels equal to, or greater than 0.2 (i.e. 3 standard deviations above mean random coherence levels) were considered significantly different from noise. Coherence values of the two frequency bands in the three areas were submitted to a three-way repeated measure ANOVA with “Time window”, “Cortical area” and “frequency band” as factors. Post-hoc tests (Holm-Sidak test corrected for multiple comparisons) were applied in case of significant effects following ANOVA. The phase of the cross spectrum was calculated for each subject at the frequency showing the highest coherence for each pair and the phase lag in radians was then transformed into milliseconds^26,49^. Since phase values are only meaningful when coherence is significant, those corresponding to regions with a mean coherence not significantly different from random were ignored.

## Electronic supplementary material

Supporting information
