# Supplementary material for: Convergence of sensory and limbic noxious input into the anterior insula and the emergence of pain from nociception
Source: Sci Rep. 2018 Sep 6;8:13360. doi: 10.1038/s41598-018-31781-z (PMC6127143; doi:10.1038/s41598-018-31781-z)
Supplement: Supplementary file 1 — Supporting information [file 41598_2018_31781_MOESM1_ESM.docx]

**Convergence of sensory and limbic noxious input into the anterior insula and the emergence of pain from nociception**

Hélène Bastuji^1,2^, Maud Frot^1^, Caroline Perchet^1^, Koichi Hagiwara^1^ and Luis Garcia-Larrea^1,3^

^1^ Central Integration of Pain (NeuroPain) Lab - Lyon Neuroscience Research Center, INSERM U1028; CNRS, UMR5292; Université Claude Bernard; Bron, F-69677, France; ^2^ Unité d’Hypnologie, Service de Neurologie Fonctionnelle et d’Épileptologie, Hôpital Neurologique, Hospices Civils de Lyon, Bron, F-69677, France; ^3^ Centre d’évaluation et de traitement de la douleur, Hôpital Neurologique, Lyon, France

Keywords: nociceptive stimulus, amygdala, insula, intracerebral EEG, human, pain matrix

**Supporting Information**

**Methods**

***Patients***

Ten patients with refractory partial epilepsy were included in the study (7 men, 3 women; mean age 27 years, range 19-51 years). To delineate the extent of the cortical epileptogenic area and to plan a tailored surgical treatment, depth EEG recording electrodes (diameter 0.8 mm; 5-15 recording contacts 2 mm long, inter-contact interval 1.5 mm) were implanted according to the stereotactic technique of Talairach and Bancaud (1). The decision to explore specific areas resulted from the observation, via video-EEG, of ictal manifestations suggesting the possibility of seizures propagating to, or originating from these regions (2) This procedure aims at recording spontaneous seizures but also includes the functional mapping of potentially eloquent cortical areas using evoked potentials recordings and cortical electrical stimulation (3,4). In agreement with French regulations relative to invasive investigations with direct individual benefit, patients were fully informed about electrode implantation, stereotactic EEG (SEEG), evoked potential recordings, and cortical stimulation procedures used to localize the epileptogenic cortical areas and gave their consent. The laser stimulation paradigm was submitted to, and approved by, the local Ethics Committee (CPP Sud Est IV n° 2006-A00572-49 and Ile de France XI n° 2017-A00464-49). Recordings were conducted after a minimal delay of five days post electrode implantation; at that time antiepileptic drugs had been tapered down (see Table 1) with daily dosages at, or slightly under, the minimum of their usual therapeutic range. None of these patients reported pain symptoms before or after the recording session.

***Electrode implantation***

Intracerebral electrodes were implanted using the Talairach’s stereotactic frame. A cerebral angiography was performed in stereotactic conditions using an X-ray source located 4.85 m away from the patient’s head. This eliminates the linear enlargement due to X-ray divergence and allows a 1:1 scale so that the films could be used for measurements without any correction. In a second step, the relevant targets were identified on the patient’s MRI, previously enlarged to a scale of one-to-one. As MR and angiographic images were at the same scale, they could easily be superimposed, so as to avoid damage to blood vessels and minimize the risk of haemorrhage during electrode implantation.

***Anatomical localization of electrode contacts***

The localization of the recording contacts was determined using 2 different procedures. In 5 patients implanted before 2010, MRI could not be performed with electrodes in place because of the physical characteristics of the stainless steel contacts. In these cases, the scale 1:1 post implantation skull radiographs performed within the stereotactic frame of Talairach and Tournoux (1988)(5) were superimposed to the pre-implantation scale 1:1 MRI slice corresponding to each electrode track, thus permitting to plot each contact onto the appropriate MRI slice of each patient and determining its coordinates [MRIcron® software; (6)]. In the other 5 patients, the implanted electrodes were MRI-compatible and cortical contacts could be directly visualized on the post-operative 3D-MRIs. In both cases, anatomical scans were acquired on a 3-Tesla Siemens Avanto Scanner using a 3D MPRAGE sequence with following parameters: TI/TR/TE 1100/2040/2.95 ms, voxel size: 1 x 1 x 1 mm^3^, FOV = 256 x 256 mm^2^.

Intracortical electrode contacts were mapped to the standard stereotaxic space (Montreal Neurological Institute, MNI) by processing MRI data with Statistical Parametric Mapping (SPM12 — Wellcome Department of Cognitive Neurology, UK; http:// www.fil.ion.ucl.ac.uk/spm/). Anatomical T1-3D images pre- and post-implantation were co-registered and normalized to the MNI template brain image using a mutual information approach (7) and the segmentation module of SPM12, which segments, corrects bias and spatially normalizes images with respect to the MNI model (8). Then, the localization of electrodes was performed directly on the MR of the patients, and using the human atlas of the insula (9) for the exact localization of the contacts within the insula. In the 5 patients with MRI-compatible electrodes, the cortical contacts could be directly visualized on the post-operative normalized 3D-MRIs. In the 5 patients without MRI-compatible electrodes, the coordinates of contacts were determined on their own MRI according to the procedure described above, thus permitting to plot each contact onto the appropriate MRI slice of each patient and determining its MNI coordinates.

***Nociceptive-specific laser stimulation.***

Radiant nociceptive heat pulses of 5 ms duration were delivered with a Nd:YAP-laser (Yttrium Aluminium Perovskite; wavelength 1.34 µm; El.En.®, Florence, Italy). The laser beam was transmitted from the generator to the stimulating probe via an optical fibre of 10 m length (550µm diameter with sub-miniature version (SAV) A-905 connector). Perceptive and nociceptive thresholds were determined in each patient immediately before the recording session. Nociceptive thresholds to A-delta stimuli were determined as the minimal laser energy producing a pricking sensation, compared to “pulling a hair” or “receiving a boiling water drop” in at least two out of three stimuli. They were obtained in all subjects with energy densities between 60 and 100 mJ/mm2 (mean 80 mJ/mm2), which are within the usual data range observed in our laboratory and those reported by others using Nd:YAP lasers (10); these parameters have been validated in humans as being able to activate the spinothalamic system without concomitant activation of the dorsal column-lemniscal system (10-12)*.*

***Data acquisition and recording procedure.***

In each patient, two runs of 10–15 stimulations each, at nociceptive threshold, were applied to the skin in the superficial radial nerve territory on the dorsum of the hand contralateral to the hemispheric side of electrodes implantation. The heat spot was slightly shifted over the skin surface between two successive stimuli to avoid both sensitisation and peripheral nociceptor fatigue. Five patients were stimulated on the right hand, and five on the left hand. Recordings were performed in common referential mode, the reference electrode being chosen for each patient on an implanted contact located in regions devoid of stimulus-evoked activity (usually skull or white matter). The EEG was recorded continuously from 96 – 128 channels, at a sampling frequency of 256 Hz or 512 Hz, then amplified and band pass filtered (0.33-128 Hz; -3dB, 12 dB/octave) to be stored in hard disk for off-line analysis (Micromed SAS®, Mâcon France).

**Electrophysiological data analyses.**

***Laser-evoked potentials (LEPs).*** The coordinates of the contacts exhibiting the largest responses to laser stimuli are indicated on Table 2. Offline analyses, including segmentation of the EEG, selective averaging, time-frequency transforms and spectral coherence analyses were performed using BrainVision® System (Brain Products®, Munich, Germany). Segments presenting contamination by epileptic transient activities or artefacts exceeding 250 µV were rejected from analysis, the rate of rejection being of ~10%. LEP components recorded in the different structures were assessed using both monopolar (referential) and bipolar montages (with adjacent contact), within a time window of 1 second (100 ms pre- and 900 ms post-stimulus). Only referential responses were analysed for this report. Were measured in each patient: (1) the onset and peak latencies of the LEP main component, and (2) its amplitude (from onset to peak). Onsets were defined at the inflection point when amplitude values of the signal differed by two standard deviations from the mean pre-stimulus baseline. Statistical analyses were performed with GraphPad Prism 6 and StatView® softwares. Latencies and amplitudes were submitted to one-way ANOVA with cortical areas as ‘between’ factor, and significance level set at p<0.05 (Greenhouse-Geisser corrected if needed). Post-hoc tests (Holm-Sidak test corrected for multiple comparisons) were applied in case of significant main effects of ANOVA.

***Functional connectivity measures.*** Intra-insular and insula-amygdala functional relationships were assessed using EEG phase-coherence between each pairs of areas (posterior insula-anterior insula, posterior insula-amygdala and anterior insula-amygdala). Phase coherence was computed after Fast Fourier Transform of the signal for each spectral band power (delta: 1-3 Hz, theta: 4-7 Hz, alpha: 8-12 Hz, beta: 13-29 Hz). The analysis was performed within three post-stimulus time windows: 100-400 ms, 400-700 ms and 700-1000 ms. The initial time window was chosen according to previous intra-cerebral recordings showing that the earliest insular responses to laser develop within these latency borders (13-16). The two other analysis windows were defined so as to divide the post-stimulus period in two equivalent segments of 300 ms each (17). Phase Coherence values were computed as the quotient between correlation and autocorrelation for each frequency and each channel pair, and underwent Fisher’s z-transformation before statistical analysis. Phase values were unwrapped (phase as a continuous signal across frequency bins), then phase angles transformed into milliseconds. Coherence values were grouped as ‘low frequencies’ (delta + theta bands, 1-7 Hz) and ‘high-frequencies’ (alpha + beta bands, 8 – 29 Hz). To determine whether a given level of coherence between two regions was above noise, its statistical significance was estimated by contrasting with random levels of coherence, obtained from recordings where the amplitude levels of one of the time series was randomly reordered (18)(e.g. Miranda de Sá et al. 2002). The resulting ‘random’ spectra showed average coherence levels of 0.042 ± 0.046 for delta + theta and 0.055 ± 0.045 for alpha + beta. Therefore, coherence levels equal to, or greater than 0.2 (i.e. 3 SDs above mean random coherence levels) were considered significantly different from noise. This level was consistent with previous studies of intracortical or scalp activity using similar recording parameters (19,20). Coherence values of the two frequency bands in the three areas were submitted to a three-way repeated measure ANOVA with “Time window”, “Cortical area” and “frequency band” as factors. Post-hoc tests (Holm-Sidak test corrected for multiple comparisons) were applied in case of significant effects following ANOVA. The phase of the cross spectrum was calculated for each subject at the frequency showing the highest coherence for each pair and the phase lag in radians was then transformed in milliseconds (21,22). After calculating the cross spectrum between two time-series representing each pair of region, phase difference (Δϕn) in radians (unwrapped) was obtained at each frequency for each trial n [1, . . ., N] and time window. Mean phase difference (Δθ) was calculated while transforming into angle: Δθ = (N-1∑ Δϕn) ×180/ π. The inter-regional conduction time in milliseconds (Δt) was then obtained in the following way: Δt = (Δθ / 360) × 103 / f, where f denotes the frequency showing the highest coherence for each time window and pair of region. Since phase values are only meaningful when coherence is significant, those corresponding to regions with a mean coherence not significantly different from random were ignored.

**References**

1. Talairach, J., Bancaud, J. Stereotactic approach to epilepsy: methodology of anatomo-functional stereotaxic investigations. *Prog. Neurol. Surg.* ***5****297-5354* (1973).

2. Guenot, M., *et al.* Neurophysiological monitoring for epilepsy surgery: the Talairach SEEG method. StereoElectroEncephaloGraphy. Indications, results, complications and therapeutic applications in a series of 100 consecutive cases. *Stereotact. Funct. Neurosurg.* **77**, 29-32 (2001).

3. Ostrowsky, K., *et al.* Representation of pain and somatic sensation in the human insula: a study of responses to direct electrical cortical stimulation. *Cereb. Cortex* **12**, 376 -385 (2002).

4. Mazzola, L., Isnard, J., Mauguière, F. Somatosensory and pain responses to stimulation of the second somatosensory area (SII) in humans. A comparison with SI and insular responses. *Cereb. Cortex* **16**, 960 -968 (2006).

5. Talairach, J., Tournoux, P. Co-planar stereotaxic atlas of the human brain: 3-dimensional proportional system: an approach to cerebral imaging. Stuttgart (Germany), Thieme (1988).

6. Rorden, C., Brett, M. Stereotaxic display of brain lesions. *Behav. Neurol.* **12**, 191-200 (2000).

7. Maes, F., *et al.* Multimodality image registration by maximization of mutual information. *IEEE Trans. Med. Imaging* **16**, 187-198 (1997).

8. Ashburner, J., Friston, K.J. Unified segmentation. *Neuroimage* **26**, 839-851 (2005).

9. Faillenot, I., Heckemann, R.A., Frot, M., Hammers, A. Macroanatomy and 3D probabilistic atlas of the human insula. *Neuroimage* **150**, 88-98 (2017).

10. Cruccu, G., *et al.* Recommendations for the clinical use of somatosensory-evoked potentials. *Clin. Neurophysiol.* **119**, 1705-1719 (2008).

11. Garcia-Larrea, L., *et al.* Operculo-insular pain (parasylvian pain): a distinct central pain syndrome. *Brain* **133**, 2528-2539 (2010).

12. Perchet, C., *et al.* Do we activate specifically somatosensory thin fibres with the concentric planar electrode? A scalp and intracranial EEG study. *Pain* **153**, 1244-1252 (2012).

13. Lenz, F.A., *et al.* Painful stimuli evoke potentials recorded from the parasylvian cortex in humans. *J. Neurophysiol.* **80**, 2077–2088 (1998).

14. Frot, M., Rambaud, L., Guenot, M., Mauguiere, F. Intracortical recordings of early pain-related CO2-laser evoked potentials in the human second somatosensory (SII) area. *Clin. Neurophysiol.* **110**, 133–145 (1999).

15. Frot, M., Faillenot, I., Mauguière, F. Processing of nociceptive input from posterior to anterior insula in humans. *Hum. Brain Mapp.* **35**, 5486-99 (2014).

16. Ohara, S., Crone, N.E., Weiss, N., Treede, R.D., Lenz, F.A. Amplitudes of laser evoked potential recorded from primary somatosensory, parasylvian and medial frontal cortex are graded with stimulus intensity. *Pain* **110**, 318–328 (2004).

17. Bastuji, H., Frot, M., Perchet, C., Magnin, M., Garcia-Larrea, L. Pain networks from the inside: Spatiotemporal analysis of brain responses leading from nociception to conscious perception. *Hum. Brain Mapp.* **37**, 4301-4315 (2016).

18. Miranda de Sá, A.M.F.L., Infantosi, A.F., Simpson, D.M. Coherence between one random and one periodic signal for measuring the strength of responses in the electro-encephalogram during sensory stimulation. *Med. Biol. Eng. Comput.* **40**, 99-104 (2002).

19. Achermann, P., Borbély, A.A. Coherence analysis of the human sleep electroencephalogram. *Neuroscience* **85**, 1195-1208 (1998).

20. Cantero, J.L., Atienza, M., Madsen, J.R., Stickgold, R. Gamma EEG dynamics in neocortex and hippocampus during human wakefulness and sleep. *Neuroimage* **22**, 1271-1280 (2004).

21. Brazier, M.A. Spread of seizure discharges in epilepsy: anatomical and electrophysiological considerations. *Exp. Neurol.* **36**, 263-272 (1972).

22. Gotman, J. Measurement of small time differences between EEG channels: method and application to epileptic seizure propagation. *Electroenceph. Clin. Neurophysiol.* **56**, 501-514 (1983).

Table SI 1. Individual clinical, MRI and SEEG data

| Patient | Gender/Age | Treatment | MRI | Seizure onset | Side of implantation |
| --- | --- | --- | --- | --- | --- |
| P1 | M/20 | None | R hippocampal atrophy | R mesial temporal | R |
| P2 | M/19 | None | Normal | R mesial temporal | R |
| P3 | M/37 | Carbamazepin 400  Topiramate 200  Clobazam 5 | Normal | L perisylvian | L |
| P4 | M/21 | Lamotrigin 100 Valproate Pregabalin | R operculo-insula dysplasia | R operculo-insula | R |
| P5 | F/51 | Oxcarbazepin clobazam | Normal | R temporal | R |
| P6 | M/20 | Valproate Carbamazepin  Levetiracetam | L temporal atrophy | L temporal | L |
| P7 | M/26 | Carbamazepin 400 | L temporal atrophy | L temporal | L |
| P8 | F/30 | Oxcarbamazepin | R temporal atrophy | R temporal | R |
| P9 | M/29 | Topiramate 75 Lamotrigin 200 Levetiracetam1000 | Normal | L temporal | L |
| P10 | F/21 | Lamotrigin 125 | Normal | L temporal | L |

Table SI 2. Values ± SEM of the mean onsets latencies, peak latencies and peak amplitudes of the responses in the three cortical areas.

|  | Onset latency (ms) | Highest peak latency (ms) | Highest peak amplitude  (µV) |
| --- | --- | --- | --- |
| Posterior insula | 137.8 ± 8.3 | 218.8 ± 7.1 | 98.7 ± 9.7 |
| Anterior insula | 161.3 ± 8.2 | 235.1 ± 10.2 | 97.3 ± 19.4 |
| Amygdala | 130.6 ± 8.1 | 425 ± 11 | 66.6 ± 6.1 |

Table SI 3. Post hoc tests following ANOVA interaction time/area of spectral coherence values. PI: posterior insula, A: amygdala, AI: anterior insula.

| time | area | t | p |
| --- | --- | --- | --- |
| 100-400 ms | PI/A vs PI/AI | 6.31 | <0.0001 |
|  | PI/A vs AI/A | 0.57 | ns |
|  | PI/AI vs AI/A | 5.74 | <0.0001 |
| 400-700 ms | PI/A vs PI/AI | 5.47 | <0.0001 |
|  | PI/A vs AI/A | 1.11 | ns |
|  | PI/AI vs AI/A | 6.59 | <0.0001 |
| 700-1000 ms | PI/A vs PI/AI | 2.43 | 0.05 |
|  | PI/A vs AI/A | 1.87 | ns |
|  | PI/AI vs AI/A | 0.56 | ns |


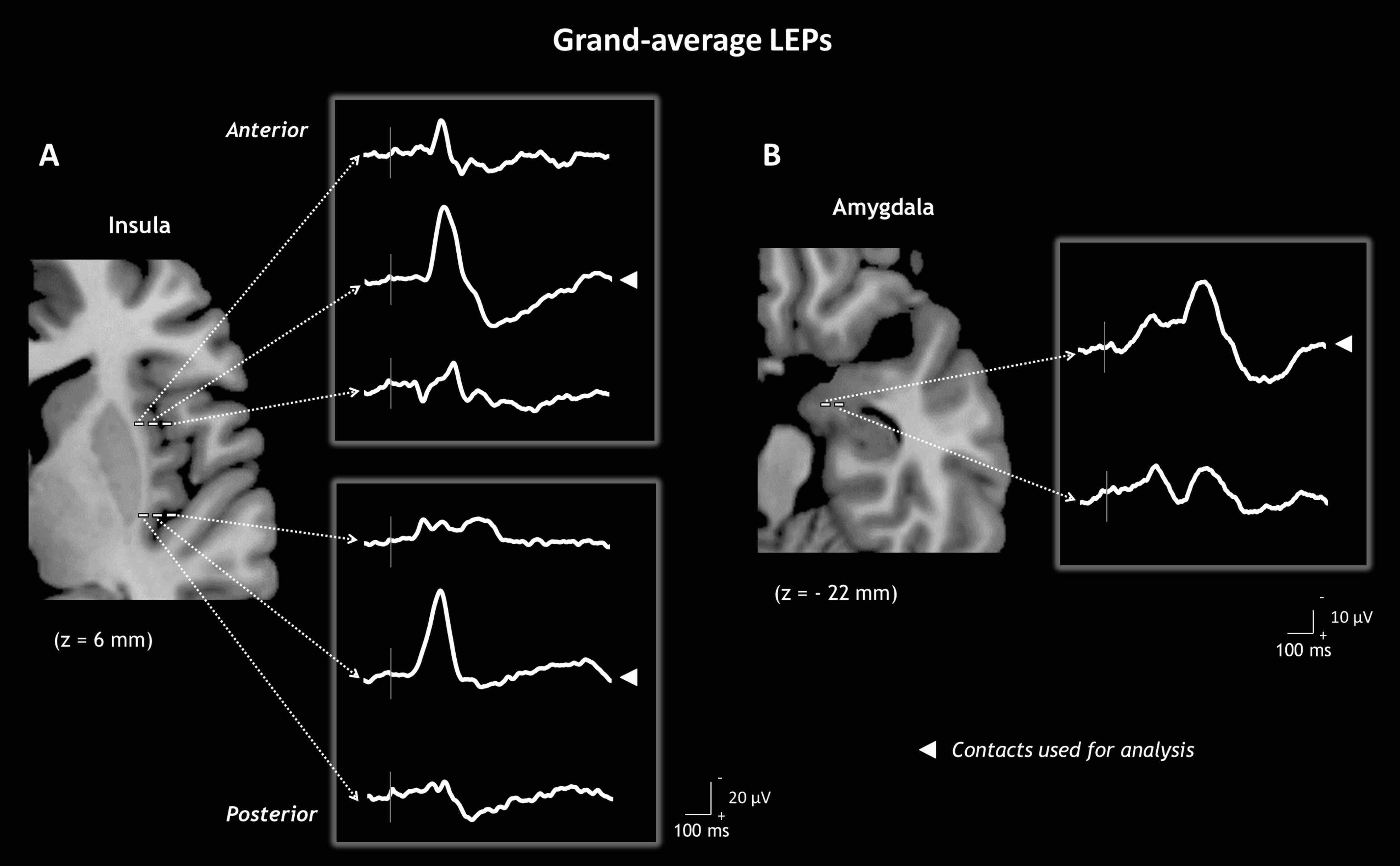


Figure S1. Laser evoked potentials (LEPs) recorded on referential mode in the posterior and anterior insulae and the amygdalar nucleus. A. Left: Three adjacent contacts of the electrodes implanted in the posterior and anterior insulae, are plotted at the mean coordinates used in this study on MNI brain template; A. Right: Grand averages of LEPs obtained in each of the 3 adjacent contacts of the electrode passing through the anterior insula (top) and the posterior insula (bottom). B. Left: Two adjacent contacts of the electrode implanted in the amygdala are plotted at the mean coordinates on MNI brain template; B. Right: Grand averages of LEPs obtained in each of the 2 adjacent contacts of the electrode passing through the amygdala.
